# A Comparison Between the Relaxation/Meditation/Mindfulness Tracker t Inventory and the Freiburg Mindfulness Inventory for Predicting General Health, Anxiety, and Anger in Adult General Population

**DOI:** 10.3389/fpsyg.2022.810383

**Published:** 2022-04-04

**Authors:** Alireza Malakoutikhah, Mohammad Ali Zakeri, Mahlagha Dehghan

**Affiliations:** ^1^Student Research Committee, School of Nursing and Midwifery, Kerman University of Medical Sciences, Kerman, Iran; ^2^Non-Communicable Diseases Research Center, Rafsanjan University of Medical Sciences, Rafsanjan, Iran; ^3^Social Determinants of Health Research Centre, Rafsanjan University of Medical Sciences, Rafsanjan, Iran; ^4^Nursing Research Center, Kerman University of Medical Sciences, Kerman, Iran; ^5^Department of Critical Care Nursing, Razi Faculty of Nursing and Midwifery, Kerman University of Medical Sciences, Kerman, Iran

**Keywords:** RMM tracker, FMI scale, general health, anxiety, anger

## Abstract

**Introduction:**

An individual’s level of mindfulness can predict his/her level of general health, anxiety, and anger. If we have a valuable tool for measuring mindfulness, we can predict such factors more concisely. Therefore, the aim of this study was to compare a narrowband and a broadband mindfulness scale in predicting the level of general health, anxiety, and anger in a general population.

**Materials and Methods:**

This was a cross-sectional study on an Iranian general population (all citizens living in Kerman) from September 22, 2020 to April 14, 2021. The convenience sampling method was used. Data were collated *via* electronic and paper forms of the Relaxation/Meditation/Mindfulness Tracker t-Persian version (RMMt-P), the Freiburg Mindfulness Inventory- Short-Form-Persian version (FMI-P), the General Health Questionnaire, the trait anxiety section of the State–Trait Anxiety Inventory, and the trait anger section of the State–Trait Anger Expression Inventory-2.

**Results:**

The FMI-P predicted 0.05% of GHQ variance while the first and third levels of RMMt-P predicted 0.145%. The FMI-P predicted 0.19% of anxiety variance, while the first and third levels of RMMt-P predicted 0.195%. The FMI-P predicted 0.0% of anger variance, while the first, second, and third levels of RMMt-P predicted 0.08%. RMMt-P Level 1 was a better predictor of general health, anger, and anxiety.

**Conclusion:**

The current study found that the RMMt-P was a better predictor of general health and anger than the FMI-P. These findings suggest that the type of questionnaire used in the study of mindfulness is important, but more research is needed to determine the extent of these relationships.

## Introduction

Mindfulness is based on eastern meditation traditions and is used as a therapeutic method to promote mental health (Desbordes et al., 2015; Rayan and Ahmad, 2018). Mindfulness is defined differently by researchers, physicians, and clinicians; each of these individuals believes that certain aspects of this concept are more important than the others (Brown et al., 2007). According to Kabat-Zinn (2012), mindfulness is having awareness, paying attention, and being in the present moment only as an observer who observes current thoughts and feelings without judging or reacting to them. Brown et al. (2009) defined mindfulness as being aware of the inputs of one’s mind and paying attention to what is happening (Brown et al., 2009). Walach et al. (2006) described it as warm, friendly, accepting, and non-judgmental attitudes toward the elements of the mind (Walach et al., 2006). Many definitions share three characteristics: being in a state of consciousness, being present in the moment, and not passing judgment (Shepherd, 2020). Mindfulness is a mental state characterized by non-judgmental thought observation that may temporarily lessen subjective information evaluation by limiting referencing to self-related values, thus possibly acts as an inhibitor of mental activities leading to anxiety, anger, or negative thoughts in general (Vuong and Napier, 2015).

Different studies have found that mindfulness-based interventions are an effective way to improve medical and psychological symptoms and conditions, as well as to improve general health, anxiety, and anger (Hirano and Yukawa, 2013; Aghaie et al., 2018; Guo et al., 2019; El Morr et al., 2020; Shepherd, 2020; Burgess et al., 2021; Takebe and Sato, 2021). The results of a systematic review and meta-analysis on the effect of mindfulness-based interventions on wellbeing, mental health, and general health showed that the mindfulness-based interventions had a large effect size on wellbeing, mental health, and general health (Aghaie et al., 2018). Karing (2021) reported that mindfulness, along with optimism, was one of the two most relevant protective factors against anxiety. In addition, Kim (2021) indicated that maladaptive emotion regulation strategies, such as rumination and expressive suppression, mediated the relationship between mindfulness and aggression and that mindfulness was associated with decreased use of these strategies. Beyond individual levels, applying mindfulness practices on larger scales requires a multifaceted integral framework. A combination of scientific research, communities’ insights, and well-coordinated management can make mindfulness an effective tool against many public health problems (Vuong et al., 2022).

Given that reviewed studies establish a relationship between mindfulness and the aforementioned variables, we could try to predict individuals’ general health, as well as their anxiety and anger levels, simply by measuring their mindfulness level. To do so, we need standard tools capable of accurately measuring this concept.

There are several tools available for measuring mindfulness with broadband and narrowband assessments of experiences. These tools vary in terms of how they measure mindfulness; some measure mindfulness as a one-dimensional structure versus a multifaceted structure (Baer et al., 2006), while others view it as a trait or state structure (Dane, 2011). Certain tools only assess a person’s mental state, while others assess feelings and physical experiences (Grossman, 2008).

The Freiburg Mindfulness Inventory (FMI) is one of the narrowband measures. Buchheld et al. (2001) developed the FMI in 2001, which consisted of 30 items divided into four factors: mindful presence, non-judgmental acceptance, openness to experience, and insight. Later, Walach et al. (2006) developed a short form of FMI (14 items), which was more suitable for use in the general population. Kohls et al. (2009)showed that the short form measured the two factors of Presence and Acceptance.

Many measures of mindfulness are narrowband measures that assess aspects of awareness, presence, and acceptance (Smith, 2019a). However, the Relaxation, Meditation, and Mindfulness Tracker t (RMMt) is known as a broadband measure to assess the 5 + 1 dimensions of Smith’s model of mindfulness and relaxation. This model is designed to measure the full range of potential mindfulness and relaxation states associated with mindfulness practices. It measures five levels of mindfulness (Mindful Basic Relaxation; Mindful Quiet Focus; Mindful Awakening; Mindful Deepening; and Mindful Transformation/Transcendence) and one dimension of Mindful Transcendent Positive Emotion (Smith, 2019a).

Mental health is a significant indicator of a society’s general health. According to World Health Organization (WHO), People with severe mental health conditions die prematurely—up to two decades earlier—due to preventable physical conditions. Mental health problems now account for one out of every five years of disability. Mental health conditions are increasing worldwide because of demographic changes. There has been a 13% rise in mental health conditions in the 10 years (2007 to 2017; World Health Organization, n. d.). Anxiety, along with depression, is one of the most common mental health conditions, costing the global economy one trillion dollars each year (World Health Organization, n. d.). Since an individual’s level of mindfulness can predict his/her level of general health, anxiety, and anger, we could predict such factors more concisely if we had a valuable tool for measuring mindfulness. Therefore, the aim of this study was to compare a narrowband mindfulness scale and a broadband mindfulness scale in predicting the level of general health, anxiety, and anger in a general population.

## Materials and Methods

### Study Design and Settings

This was a cross-sectional study to compare two measures of mindfulness (i.e., the RMMt-P and FMI-P) in predicting the general health, anxiety, and anger of an Iranian general population from September 22, 2020 to April 14, 2021.

### Participants, Sampling, and Sample Size

The study population consisted of all residents of Kerman City in southeast Iran. The study sample consisted of all citizens living in Kerman City who met the inclusion criteria. The following were among the inclusion criteria: (1) participants must be at least 18 years old, (2) participants must have basic reading and writing skills, and (3) participants must not have any psychiatric disorders. Exclusion criteria were met if more than 10% of the questions on each questionnaire were not completed.

The convenience sampling method was used. Questionnaires were distributed in both electronic and paper formats. The electronic form was distributed *via* email or popular Iranian social networks (WhatsApp, Telegram, etc.). For the paper form, we divided the city into four districts according to the municipal divisions, and each district was treated as a cluster. Then, shopping malls, parks, recreation areas, and streets were considered as the research settings.

Using the Cochran’s formulas, 384 samples were estimated (*α* = 0.05, *d* = 0.05, *Z* = 1.94). Considering dropouts, 470 questionnaires were distributed. Twelve questionnaires were excluded from the study regarding confounding information and missing values. Finally, 458 questionnaires were subjected to analysis.

### Measurements

#### Demographic Characteristics Form

Demographic Characteristics Form consisted of questions about age, gender, marital status, educational level, occupation, income, prior knowledge of the mindfulness concept (yes/no), and use of any methods of mindfulness (yes/no).

#### Relaxation/Meditation/Mindfulness Tracker t-Persian Version

The RMM Trackers are a series of broadband self-report inventories of relaxation, meditation, and mindfulness. The RMM Tracker t (trait) is a dispositional or trait inventory used to assess how often one experiences different RMM states. It consisted of 25 specific RMM states divided into five levels of mindfulness (Mindful Basic Relaxation; Mindful Quiet Focus; Mindful Awakening; Mindful Deepening; and Mindful Transformation/Transcendence) and one dimension of Mindful Transcendent Positive Emotion.

The RMM Tracker t items are scored on a 13-point Likert scale (never/do not understand item = 0, once a year = 3, once a month = 6, once a week = 9, and about every day = 12). The item scores of each level are added together and divided by the number of items in that level. The higher the score in each level, the higher and more positive the level of that characteristic (Smith, 2019a).

The Persian version of RMMt consists of 23 RMM states. There are 23 specific RMM states, which are divided into three dynamic and interacting levels. Level 1—Mindful Love, Thankfulness, and Transcendence includes the RMM states of loving, caring, compassion, thankful, sense of something greater, prayerful, clear, awake, aware, happy, optimistic, beautiful, trusting, fantasy, interested, curious, fascinated, meaning, purpose, and direction (10 items). Level 2—Relaxation includes the RMM states of being unbothered, easy, effortless, quiet, refreshed, at ease, at peace, far away, physically relaxed, pleasant mind wandering, centered, and grounded (9 items). Level 3—Mindful Deepening includes the RMM states of going deeper, spaciousness, expansiveness, observer, and “spiritual” or “mystical” (4 items). The content and construct validities of the Persian version of RMMt have been confirmed. In addition, the Cronbach’s alpha values of the three levels were 0.93, 0.91, and 0.80, respectively (Malakoutikhah et al., 2021).

#### The Freiburg Mindfulness Inventory-Short-Form-Persian Version

FMI has proven to be an effective psychometric tool for assessing mindfulness in both clinical and non-clinical populations. Buchhold et al. designed the FMI in 2001, which consisted of 30 items. Later, Walach et al. developed the short form (14 items: with two dimensions of the presence and the acceptance) which was more suitable for use in the general population. The FMI-SF items are scored on a 4-point Likert scale (rarely = 1 to almost always = 4). The minimum and maximum scores are 14 and 56, respectively. A higher score indicates that you are more mindful (Walach et al., 2006).

Ghasemi Jobaneh et al. found that in an Iranian population, the validity and reliability of FMI-SF were acceptable. In addition, according to the confirmatory factory analysis, the Persian version of FMI-SF was unidimensional (GhasemiJobaneh et al., 2015).

#### The General Health Questionnaire

The GHQ is used to measure psychological distress in a variety of settings. It is a 60-item self-report questionnaire. There are shorter forms in 12, 20, 28, and 30 items. The GHQ assesses an individual’s mental state in the past month. The GHQ-12 consists of 12 items, six of which are positive and six of which are negative. The items are scored using a 4-point Likert scale (all items coded as 0-1-2-3). The minimum and maximum scores would be 0 and 36, respectively. The higher the score, the higher the psychological distress (Goldberg, 1972, 1988).

Namjoo et al. found that the content validity index and content validity ratio of the GHQ-12 in an Iranian population were 0.92 and 0.96, respectively. Cronbach’s alpha coefficients were also reported to be 0.82 (Namjoo et al., 2017) and 0.85 by Rahmati et al. (Najarkolaei et al., 2014).

#### The Trait Anxiety Section of the State–Trait Anxiety Inventory

Spielberger et al. developed STAI in 1970 as tool for measuring trait–state anxiety. This questionnaire includes two separate self-assessment scales, each with 20 items, for a total of 40 items. The trait scale measures an individual’s general and normal emotions. All items are scored on a 4-point Likert scale from “1 = Not at All” to “4 = Very Much” with higher scores indicating greater anxiety (Spielberger et al., 1983a).

Rabiee et al. (2007) reported the reliability of the STAI as 0.89. In addition, Mehram reported the reliability of STAI based on Cronbach’s alpha coefficients as 0.90 and the validity for state and trait anxiety as 0.95 and 0.99, respectively (Mahram, 1993).

#### The Trait Anger Section of the State–Trait Anger Expression Inventory-2

Spielberger et al. designed STAXI in 1983 to assess the severity of different states of anger (Spielberger et al., 1983b). Based on studies from 1995 to 1999, STAXI was changed and revised into STAXI-2. The STAXI-2 was designed for two purposes: (1) evaluating anger factors in order to distinguish between normal and abnormal personality; (2) providing averages of various anger factors that contribute to medical problems. The STAXI-2 consists of 57 items divided into three sections. Each section measures state anger, trait anger, and anger expression and control. Each item is scored on a four-point Likert scale (Spielberger, 1999).

Asghari et al. (2008) reported the internal consistency coefficients of STAXI-2 ≥ 0.73 in university students based on Cronbach’s alpha coefficients. Khodayarifard et al. reported the reliability of STAXI-2 based on Cronbach’s alpha coefficients ranging from 0.60 to 0.93; for the trait anger section, Cronbach’s alpha coefficients was 0.83 (Khodayari-Fard et al., 2010).

### Data Collection and Data Analysis

Google forms were used to create and distribute the questionnaire’s online form. Ten participants checked the online form in terms of resolving the problems. Nearly 160 participants completed the online form and the rest answered the paper form.

SPSS version 22 was used for data analysis. Frequency, percentage, mean, and standard deviation were used to describe the participants’ characteristics. Pearson correlation coefficients were used to determine the correlation between RRMt-P, FMI-P, GHQ, anxiety, and anger scores because their distributions were normal. Multiple linear regression with stepwise method was used to determine the power of RMMt-P and FMI-P in predicting GHQ score, anxiety, and anger. In addition, as the Persian version of FMI is unidimensional, we only used the total score for predicting the GHQ, anxiety, and anger scores. A significance level of 0.05 was considered.

## Ethical Consideration

The code of ethics was obtained from the Ethics Committee of Kerman University of Medical Sciences (Ethical Code: IR.KMU.REC.1398.673). In addition, Dr. Smith authorized the translation and use of RMM. Before the inclusion of participants into the study, the researcher presented them with a consent form that included the following information: (1) the study purpose and objectives; (2) the information confidentiality; and (3) the anonymous participants, who can withdraw from the study at any time.

## Results

The mean age of the participants was 34.37 ± 10.79 year. The majority of the participants was female, married, employed, and had academic education. The majority of the participants had no knowledge of mindfulness and did not use any methods of mindfulness.

### Descriptive Statistics for Main Study Variable

The mean scores for the first, second, and third levels of RMMt-P were 7.04, 7.08, and 6.72, respectively, all of which were higher than the scale’s midpoint of 6. The mean score of the FMI-P was 32.56, which was lower than the scale’s midpoint of 35. The mean score of the GHQ was 14.17, which was less than the scale’s midpoint of 18. The mean score of the anxiety was 48.76, which was less than the scale’s midpoint of 50. The mean score of the anger was 23.36, which was less than the scale’s midpoint of 25 (Table 1).

**Table 1:** Summary of descriptive statistics for main study variables (*n* = 458).

Variable		Mean	Standard deviation
General health		14.17	4.28
Anxiety		48.76	6.16
Anger		23.36	4.44
FMI-P-total score		32.56	4.68
RMMt-Persian scales	RMMt-P level 1—mindful love, thankfulness, and transcendence	7.04	2.08
	RMMt-P level 2—relaxation	7.08	2.02
	RMMt-P level 3—mindful deepening	6.72	2.21

### Correlation Coefficient Matrix

The FMI-P score had a significant negative correlation with general health and anxiety but not with anger. RMMt-P levels 1 and 2 had significant negative correlations with general health, anxiety, and anger. The third level of RMMt-P had a significant negative correlation with general health, and anxiety but not with anger (Table 2).

**Table 2:** Correlation coefficient matrix for main study variables.

Variable		General health	Anxiety	Anger
FMI-P-total score		−0.24(<0.001)	−0.44(<0.001)	0.02(0.70)
RMMt-Persian scales	RMMt-P level 1—mindful love, thankfulness, and transcendence	−0.37(<0.001)	−0.42(<0.001)	−0.23(<0.001)
	RMMt-P level 2—relaxation	−0.29(<0.001)	−0.34(<0.001)	−0.10(0.03)
	RMMt-P level 3—mindful deepening	−0.20(<0.001)	−0.22(<0.001)	−0.07(0.15)

### Multiple Regression

The FMI-P predicted 0.05% of GHQ variance while the first and third levels of RMMt-P predicted 0.145%. RMMt-P Level —Mindful Love, Thankfulness, and Transcendence, was a better predictor of general health (Table 3).

**Table 3:** Comparison of the linear regression models of RMMt-P and FMI-P for predicting general health.

Variable		B	SE	*β*	*t*	*P*	95% CI for B	Adjusted *R*^2^
General Health	Constant	21.18	1.37		15.50	<0.001	18.49–23.86	0.05^*^
	FMI-P-total score	−0.22	0.04	−0.24	−5.18	<0.001	−0.30 – –0.13	
	Constant	19.03	0.68		28.18	<0.001	17.71–20.36	0.145^**^
	RMMt-P level 1- mindful love, thankfulness, and transcendence	−0.10	0.01	−0.49	−7.64	<0.001	−0.13 – 0.08	
	RMMt-P level 3 - mindful deepening	0.08	0.03	0.17	2.59	0.01	0.02–0.15	

*SE, Standard error; CI, Confidence intervals*.

* *F = 26.83, p < 0.001*;

** *F = 39.63, p < 0.001*.

The FMI-P predicted 0.19% of anxiety variance, while the first and third levels of RMMt-P predicted 0.195%. RMMt-P Level 1—Mindful Love, Thankfulness, and Transcendence was a better predictor of anxiety (Table 4).

**Table 4:** Comparison of the linear regression models of RMMt-P and FMI-P for predicting anxiety.

Variable		B	SE	*β*	*t*	*P*	95% CI for B	Adjusted *R*^2^
Anxiety	Constant	67.70	1.82		37.29	<0.001	64.13–71.26	0.19^*^
	FMI-P-total score	−0.58	0.06	−0.44	−10.54	<0.001	−0.69 – –0.47	
	Constant	56.72	0.94		60.10	<0.001	54.86–58.57	0.195^**^
	RMMt-P level 1- mindful love, thankfulness, and transcendence	−0.17	0.02	−0.58	−9.26	<0.001	−0.21 – –0.14	
	RMMt-P level 3 - mindful deepening	0.15	0.04	0.21	3.37	0.001	0.06–0.24	

*SE, Standard error; CI, Confidence intervals*.

* *F = 111.09, p < 0.001*;

** *F = 56.40; p < 0.001*.

The FMI-P predicted 0.0% of anger variance, while the first, second, and third levels of RMMt-P predicted 0.08%. RMMt-P Level 1—Mindful Love, Thankfulness, and Transcendence was a better predictor of anger (Table 5).

**Table 5:** Comparison of the linear regression models of RMMt-P and FMI-P for predicting anger.

Variable		B	SE	*β*	*t*	*P*	95% CI for B	Adjusted *R*^2^
Anger	Constant	22.81	1.46		15.63	<0.001	19.94–25.67	0.00^*^
	FMI-P-total score	0.02	0.04	0.02	0.38	0.70	−0.07 – 0.10	
	Constant	25.68	0.76		33.78	<0.001	24.19–27.17	0.08^**^
	RMMt-P level 1- mindful love, thankfulness, and transcendence	−0.10	0.02	−0.48	−5.95	<0.001	−0.14 – –0.07	
	RMMt-P level 2 – relaxation	0.09	0.04	0.17	2.40	0.02	0.02–0.16	
	RMMt-P level 3 - mindful deepening	0.04	0.02	0.16	2.01	0.045	0.001–0.08	

*SE, Standard error; CI, Confidence intervals*.

* *F = 0.14, p = 0.70*;

** *F = 13.45; p < 0.001*.

## Discussion

The purpose of this study was to compare the two scales, RMMt-P and FMI-P, for predicting general health, anxiety, and anger in an Iranian adult population. Mindfulness is a concept that emphasizes the significance of self-consciousness and refers to an immediate experience in the present moment as well as a non-judgmental approach to the mind’s process. Mindfulness denotes an increase in awareness of all mental contents, including cognition, perception, physical sensation, and so on (Baer, 2011).

Many communities in the fields of psychology and psychiatry have considered mindfulness in recent years. Following the publication of studies on mindfulness-based intervention (MBI; Hulsbosch et al., 2020) and mindfulness-based stress reduction (MBSR; Thomas et al., 2017), there was an increase in research in this area. Researchers’ attention has been drawn to practical and effective tools in order to measure and evaluate mindfulness. Various scales in the literature assess mindfulness processes in various ways. The Freiburg Mindfulness Inventory (FMI), for example, indicates a broad understanding of mindfulness. The FMI30 (the first published version of the FMI) was discovered to assess the level of mindfulness from various perspectives (Walach et al., 2006). In a general population without a history of meditation, a short version of FMI (14 items) was studied, and it was discovered that FMI14 assessed mindfulness as a one-dimensional and narrowband structure (Walach et al., 2006).

Smith developed the RMM Tracker t as a broadband scale to assess the dimensions of mindfulness across the full range of mindfulness and relaxation modes, while the RMM 25 measures relaxation, meditation, and mindfulness (Smith, 2021). When compared to questionnaires that assess mindfulness in one-dimensional and narrowband structures, such as FMI, it measures mindfulness in more dimensions with a broadband scale. However, research has shown that mindfulness is one of the potential health mediators. According to Keng et al. (2011) mindfulness has positive psychological effects such as increasing subjective wellbeing, reducing psychological symptoms and emotional reactivity, and regulating and improving various behaviors associated with mental health and higher levels of life satisfaction. The majority of aspects of mindfulness was related to the experience of meditation as well as psychological symptoms (Malakoutikhah et al., 2021) and wellbeing (Baer et al., 2008). These findings in mindfulness are consistent with the findings of the current study. The RMMt-P and FMI-P scales were found to be positively correlated with general health and negatively correlated with anxiety in the current study.

In a review of the literature, mindfulness as measured by the narrowband scale of FMI was found to be predictive of general health. Dashti et al. used the FMI to study mindfulness and discovered that quality of life, physical, mental, and social health improved in cardiovascular patients who were more mindful (Dashti et al., 2018). Dehghan et al. used the FMI to assess mindfulness in cancer patients and discovered that higher levels of mindfulness were associated with higher quality of life and lower perceived stress (Dehghan et al., 2020). According to Asgari and Shafiee (2017), measuring mindfulness with the FMI predicted 24 percent variances in quality of life of the older people, and increasing mindfulness increased quality of life of the older people.

We could not find a study that looked at the effect of RMMt on general health because of its novelty. The Five Facet Mindfulness Questionnaire (FFMQ), a broadband mindfulness questionnaire, includes five skills of observing, describing, acting with awareness, non-judging inner experience, and non-reactivity to inner experience, which may predict psychological symptoms and wellbeing (Baer et al., 2008). According to Kabat-Zinn theory (Kabat-Zinn, 2013), increased self-awareness as a result of mindfulness aids in the balance of positive and negative emotions, coping strategies for dealing with life challenges, mood, and stress management, and thus leads to an increase in people’s emotional and social dimensions. Mindfulness possibly acts as an inhibitor of mental activities leading to anxiety, anger, or negative thoughts in general (Vuong and Napier, 2015).

Furthermore, mindfulness allows people to perceive their lives more effectively and to feel more at ease with themselves. Such cognitive and emotional changes can aid in the improvement of people’s health.

The current study found that the RMMt-P and FMI-P were both negatively related to anxiety. Consistent with the current study’s findings, Hulsbosch et al. (2020) used the FFMQ, a broadband mindfulness questionnaire similar to the RMM, and demonstrated that mindfulness could reduce distress in pregnant women. Furthermore, Navarro-Haro et al. found that it reduced anxiety in patients with anxiety symptoms and generalized anxiety disorder (GAD; Navarro-Haro et al., 2019) as well as social anxiety disorder (Koszycki et al., 2016).

Dashti et al. used FMI as a narrowband scale to assess mindfulness and discovered that cardiovascular patients who were more mindful had lower levels of depression, anxiety, and stress (Dashti et al., 2018). These results have also been confirmed in patients under hemodialysis (Dehghan et al., 2021b). Furthermore, Conversano et al. showed that using the MAAS as a narrowband scale to assess mindfulness was the best predictor of psychological distress in people with COVID-19 disease. As a result, mindfulness training has the potential to be an effective intervention in preventing the onset of post-traumatic stress disorder and the occurrence of chronic mental disorders (Conversano et al., 2020).

In contrast to the current study’s findings, Dehghan et al. (2021a) showed that the study of mindfulness with the FMI scale was not associated with COVID-19 anxiety in cancer patients. However, high levels of mental and physical anxiety, as well as concern about COVID-19, have been observed in cancer patients, causing difficulties in their lives (Dehghan et al., 2021b). The conditions caused by COVID-19 disease, which can cause high levels of anxiety and stress in people (Zakeri et al., 2021), may affect the findings of Dehghan et al. (2021b). Therefore, additional research is required, particularly in critical situations. Furthermore, Blanck et al. (2018) found that regular and simple mindfulness-based interventions were beneficial even when not integrated into larger therapeutic frameworks; however, it had small and moderate effects on anxiety (Blanck et al., 2018). Blanck et al., however, did not specify the type of questionnaire used to assess mindfulness. Due to the broad concept of mindfulness, the current study focused on the type of mindfulness questionnaire (narrowband and broadband), which is significant and unprecedented. Therefore, given the variable range of mindfulness questionnaires, special consideration should be given to the type of questionnaire in future studies.

According to a review of the literature, mindfulness is an effective intervention and treatment for many conditions, including stress, anxiety, and depression, regardless of the type of questionnaire (narrowband and broadband). According to the findings, the Short-Form Mindfulness-Based Stress Reduction can help reduce individuals’ anxiety and improve quality of life and mental health (Smith et al., 2015). Increasing mindfulness through a Mindfulness-Based Cognitive Therapy (MBCT) program improves psychological wellbeing, psychopathology, and anxiety and concern (Ruths et al., 2013). Some studies have shown that mindfulness can be considered an effective tool against many public health problems (Vuong et al., 2022).

The current study found that the RMMt-P had a negative correlation with anger, whereas the FMI-P scale did not. Consistent with the findings of the current study, Světlák et al. (2021) measured mindfulness in students using the FFMQ as a broadband scale and found that mindfulness reduced perceived stress, the frequency and severity of negative effects, and increased self-compassion (Světlák et al., 2021), all of which can reduce people’s anger.

According to the current study’s findings, measuring mindfulness with the Mindful Attention Awareness Scale (MAAS) as a one-dimensional and narrowband scale is negatively related to general aggression, physical aggression, anger, and self-harm in adults (Yusainy and Lawrence, 2014). Zubair et al. (2018) used the MAAS to assess mindfulness and found a positive relationship between mindfulness and mental wellbeing in Pakistani and Russian students. These findings indicate that people who are more mindful and aware of their surroundings have better problem-solving skills, implying that mindfulness can adjust people’s mental performance and reduce anger.

According to the findings of some studies, when a person can understand his thoughts without passing judgment or reacting to them, he achieves a state of comfort and relaxation in unpleasant situations. Relaxing in an unpleasant situation reduces stress and promotes wellbeing. Mindfulness alleviates the psychological and physical symptoms of anxiety by relaxing and assists the individual in overcoming anger by adopting a new perspective, focusing on the source of stress, reducing anxiety, increasing stress resistance, and coping skills (Kabat-Zinn, 2013). This finding may point to a relationship between mindfulness and a correct understanding of our interactions and behaviors with others. Shepherd discovered that mindfulness improved people’s reflection on interpersonal behaviors and attitudes toward themselves and others. Mindfulness promotes honest reflection on issues, onset of positive actions regarding people’s behavior, and assists them in understanding how to behave with others (Shepherd, 2020).

In the current study, only RMMt-P Level 1 (Mindful Love, Thankfulness, and Transcendence) was found to be the best predictor of anxiety, general health, and anger when compared to RMMt-P and FMI-P. Smith (2019a) pointed to four specific cases in the RMM level 1 “Mindful Basic Relaxation” that all aim to reduce aversive stimulation, improve general health, and alleviate anxiety and anger. RMM 1 includes “Far Away.” The practicer feels detached from the stresses of everyday life and free of anxiety and concern. RMM 2 is “Physically Relaxed,” which refers to experiences of decreased muscle tension and increased breathing relaxation. RMM 3 “At Ease/At Peace” refers to the release of stress or mental distress. When the practicer feels relaxed again, RMM 4 “Refreshed” occurs. RMM 10 (Unbothered) also refers to a judgment-free attitude, which involves lowering one’s judgment about negative thoughts or feelings that can cause anxiety and concern (Smith, 2019b).

According to Davis and Hayes (2011), mindfulness and meditation have numerous benefits, including improved emotional skills such as emotion regulation, decreased reactivity, increased flexibility and processing speed, and increased self-insight, morality, intuition, and fear management (Davis and Hayes, 2011). According to the findings of this study, mindfulness practice may be beneficial to people’s health. Understanding and measuring these experiences, on the other hand, are related to various dimensions of mindfulness states that should be taken into account in future research. Analyzing questionnaires and paying attention to the type of questionnaire used to discover different dimensions of mindfulness might also be beneficial in order to understand mindfulness better.

In comparing the predictive power of the RMMt-P and FMI-P scales, the current study found that the RMMt-P was a better predictor of general health and anger than the FMI-P. However, no difference in anxiety prediction was found between the RMMt-P and FMI-P scales. According to a review of the literature, the relationship between mindfulness and the FMI-P as a narrowband scale predicted 15% of variances in quality of life (Dehghan et al., 2020) and 24% of variances in quality of life of older people (Asgari and Shafiee, 2017).

RMMt did not have a comparable study. The FFMQ, a broadband mindfulness questionnaire similar to RMMt, could show 16 percent variances in quality of life of patients with multiple sclerosis (MS; Schirda et al., 2015) and predict 27 percent variances in quality of life of cancer survivors living with chronic neuropathic pain (Poulin et al., 2016). These findings suggest that the FFMQ, like the RMMt, has better predictability as a broadband mindfulness questionnaire.

According to Geise’s (2019) study, it is importance to use broadband mindfulness measures that assess for other facets of mindfulness, like transcendence and fantasy, outside of the traditional scales of presence, acceptance, and awareness. Using a narrowband mindfulness questionnaire gives a one-factor mindfulness score. Given the diverse range of experiences that can be associated with mindfulness, the use of a single-factor score may miss some important levels and characteristics of mindfulness. Factors that are actually related to mindfulness but due to the lack of attention may have limited the possibility of finding more accurate patterns in mindfulness (Geise, 2019). Given that the assessment of mindfulness is still in its infancy, supplementary item-level analyses may prove to be fruitful as well.

However, a review of the findings reveals that, in addition to the type of mindfulness questionnaires used, the type of patients and the type of quality of life questionnaire (multiple choice) used can all have an impact on the findings. We also could not find a study on the RMMt scale, so we turned to similar broadband mindfulness questionnaires (FFMQs), which can help interpret the results. Another point to consider is that we did not find a study on general health and instead used quality of life results, which have health dimensions that should be considered in future studies. The current study is the first of its kind, and because there have not been any other studies like it, further discussion in this area is not possible. As a result, future research should examine and compare two types of narrowband and broadband mindfulness questionnaires in terms of predicting general health, anxiety, and anger.

As a formal statement of shortcomings should keep authors and the public from overstating a study’s claims (Vuong, 2020), our study has a number of limitations which should mentioned. First, the questionnaires’ self-report nature limited the results and may have influenced the results of specific evaluations. Second, there are few studies on the RMMt as broadband mindfulness questionnaires. Third, the current study is one of the first to review and compare narrowband and broadband mindfulness questionnaires, according to the literature review. As a result, future research should consider factors influencing results, such as study time and target population. In the present study, most of the participants were female, employed, and married, this may raise the possibility that conclusions from this population are not acceptable to other groups in the other regions. Therefore, care must be taken in interpreting the results. In the present study, the FMI questionnaire has been used to compare with RMM for predicting the variables. It is suggested that other similar questionnaires such as FFMQ be used in future studies. Since only two of the tools were compared, the conclusion should be interpreted with caution. Our study describes the comparison between these two questionnaires (RMM and FMI) to predict some health-related issues. However, it is not possible to say with certainty that better prognosis actually leads to clinical benefit, so care should be taken in interpreting the results and considering them in future studies.

## Conclusion

The current study looked at RMMt-P and FMI-P to see if they could predict general health, anxiety, and anger in the general adult population. RMMt-P and FMI-P both predicted general health and anxiety, indicating a relationship between mindfulness, general health, and anxiety as measured by the narrowband and broadband mindfulness questionnaires. Furthermore, as a broadband mindfulness questionnaire, only the RMMt-P predicted anger. The present study founded that RMMt-P may be a better predictor of general health and anger than FMI-P. However, no difference in anxiety prediction was found between the RMMt-P and FMI-P. However, due to the scarcity of comparable studies in this area, future research should review and compare the narrowband and broadband mindfulness questionnaires in predicting general health, anxiety, and anger. These findings suggest that the type of questionnaire used in the study of mindfulness is important, but more research is needed to determine the extent of these relationships.

## Data Availability Statement

The raw data supporting the conclusions of this article will be made available by the authors, without undue reservation.

## Ethics Statement

The studies involving human participants were reviewed and approved by the Ethics Committee of Kerman University of Medical Sciences (Ethical Code: IR.KMU.REC.1398.673). The participants provided their written informed consent to participate in this study.

## Author Contributions

AM, MZ, and MD designed the study. AM and MZ wrote the manuscript. MD provided critical feedback on the study and statistical analysis, and inputted to the draft of this manuscript. AM collected the data. All authors contributed to the article and approved the submitted version.

## Conflict of Interest

The authors declare that the research was conducted in the absence of any commercial or financial relationships that could be construed as a potential conflict of interest.

## Publisher’s Note

All claims expressed in this article are solely those of the authors and do not necessarily represent those of their affiliated organizations, or those of the publisher, the editors and the reviewers. Any product that may be evaluated in this article, or claim that may be made by its manufacturer, is not guaranteed or endorsed by the publisher.
